# Supplementary material for: Distribution and establishment of the alien Australian redclaw crayfish, Cherax quadricarinatus, in South Africa and Swaziland
Source: PeerJ. 2017 Apr 19;5:e3135. doi: 10.7717/peerj.3135 (PMC5399870; doi:10.7717/peerj.3135)
Supplement: Table S1 [file peerj-05-3135-s003.pdf]

Site	Coordinates	Location	Country	Elevation (m)
**K07**	25°56'01.0"S- 31°39'34.0"E	Komati River	Swaziland	274
**K08**	26°01'57.6"S -31°35'22.4"E	Komati River	Swaziland	307
**K09**	26°06'00.4"S- 31°30'58.9"E	Komati River	Swaziland	320
**LT01**	25°36'22.5"S -31°35'53.5"E	Tributary Lomati River	South Africa	292
**Driekoppies Dam**	25°42'39.6"S- 31°31'00.9"E	Dam on the Lomati River	South Africa	330
**L04**	25°45'22.3"S -31°27'13.1"E	Lomati River	Swaziland	343
**L05**	25°49'34.2"S -31°22'46.6"E	Lomati River	Swaziland	433
**C01**	25°23'28.2"S -31°58'28.4"E	Crocodile River	South Africa	133
**C02**	25°18'54.1"S -31°44'58.2"E	Crocodile River	South Africa	200
**C03**	25°22'44.8"S -31°42'28.6"E	Crocodile River	South Africa	218
**MB03**	26°05'27.2"S - 31°49'20.4"E	Mbuluzi River	Swaziland	225
**MBT01**	26°05'01.1"S- 31°50'35.3"E	Tributary Mbuluzi River	Swaziland	225
**US03**	26°51'01.9"S-32°12'21.9"E	Usutu River	South Africa	41
**US04**	26°44'46.4"S -31°48'23.8"E	Usutu River	Swaziland	150
**ND01**	26°53'16.5"S-32°17'54.8"E	Pan in Ndumo Game Reserve	South Africa	31
**ND02**	26°52'22.8"S-32°16'17.7"E	Tributary Ndumo Game Reserve	South Africa	35
**ND03**	26°51'36.4"S-32°15'31.0"E	Tributary Ndumo Game Reserve	South Africa	33
**PG01**	27°23'26.2"S-32°08'23.3"E	Pongola River	South Africa	68
**PG02**	27°23'19.7"S -31°50'16.0"E	Pongola River	South Africa	147
**Pongolapoort Dam**	27°25'13.2"S-32°03'57.1"E	Dam on the Pongola River	South Africa	155
**RB01**	28°44'16.7"S-32°04'08.6"E	Wetland in Richard's Bay area	South Africa	21
**RB02**	28°45'36.7"S-32°04'44.3"E	Lake in Richard's Bay area	South Africa	7
**Goedertrouw Dam**	28°46'30.5"S-31°28'04.9"E	Dam in KwaZulu-Natal	South Africa	218
**Albert Falls Dam**	29°25'42.17"S-30°25'31.33"E	Dam in KwaZulu-Natal	South Africa	659
