# Supplementary material for: Distribution and establishment of the alien Australian redclaw crayfish, Cherax quadricarinatus, in South Africa and Swaziland
Source: PeerJ. 2017 Apr 19;5:e3135. doi: 10.7717/peerj.3135 (PMC5399870; doi:10.7717/peerj.3135)
Supplement: Table S2 [file peerj-05-3135-s004.pdf]

Site	Month	Size female (mm)	Mass female (g)	N eggs	N newly hatched
**K01**	December	64	68	454	
**K02**	October	79	98	281	
**K03**	October	57	40	311	
**D01**	October	64	56	368	
**D01**	December	-	-	539	
		64	54		20
**D02**	October	55	36		18
